# ONLINE DEMENTIA BOOTCAMP FOR HIGH SCHOOL STUDENTS

**DOI:** 10.1093/geroni/igad104.0472

**Published:** 2023-12-21

**Authors:** Beth Mastel-Smith, Michelle Kimzey, Jennifer Garner

**Affiliations:** The University of Texas at Tyler, Tyler, Texas, United States; Texas Christian University (TCU), Fort Worth, Texas, United States; The University of Texas at Tyler, Tyler, Texas, United States

## Abstract

Caring for people with dementia requires specialized competencies. Previous education programs had a positive effect on college students’ dementia knowledge, communication skills, empathy for and attitudes towards people with dementia. To prepare high school students to care for people with dementia – whether it be in a professional or personal capacity - a six session online Dementia Bootcamp was created. Topics included dementia overview, prevention, symptoms, and a framework for understanding them, communication, family support, and evaluation. Information was delivered via short didactic instruction, presentations by people with dementia, and experiential activities such as a virtual dementia simulation. Students met with a mentor, a person with dementia, or volunteered at a dementia day program. Parental consent and student assent were obtained for six students who participated in a focus group. The focus group was audio recorded and an interview schedule was used to understand what students learned, their response to online delivery, and recommendations for future offerings. Data was transcribed and verified, coded for categories and themes developed. Increased dementia awareness and knowledge, confidence in the ability to communicate with someone with dementia, how to prevent dementia, improved cognitive empathy and empathic imagination, and reduced stigma were reported. Online delivery was well received; students recommended longer sessions, more engagement with content and each other, and more information about how dementia affects the brain. The program has been integrated into a high-school health sciences curriculum.

